# A subset of high‐grade sarcomas with myogenic differentiation are associated with recurrent *FGFR* fusions

**DOI:** 10.1002/2056-4538.70094

**Published:** 2026-05-25

**Authors:** Maximus CF Yeung, Carla Saoud, Sarah Chiang, Meera Hameed, Cristina R Antonescu

**Affiliations:** ^1^ Department of Pathology, School of Clinical Medicine, LKS Faculty of Medicine The University of Hong Kong, Queen Mary Hospital Hong Kong; ^2^ Department of Pathology and Laboratory Medicine Memorial Sloan Kettering Cancer Center New York NY USA

**Keywords:** sarcoma, FGFR, fusion, myogenic

## Abstract

Recurrent fusions involving *FGFR1‐4* genes have been previously described in rare subsets of mostly benign chondroid and mesenchymal neoplasms involving bone and soft tissue. However, a more comprehensive analysis of sarcomas associated with *FGFR* fusions, including their incidence and histotypes, has not been performed. Triggered by an *FGFR1*‐rearranged unclassified high‐grade sarcoma with myogenic differentiation, we investigated our molecular database for sarcomas with *FGFR* gene fusions to assess their recurrent potential and morphologic spectrum. A total of six unclassified sarcomas were identified, occurring in five females and one male, with a median age of 66.5 years. Tumors were located in the uterus and retroperitoneal/trunk soft tissue. Histologically, all tumors were high grade, composed predominantly of spindle cell morphology with variable cytologic atypia, high mitotic activity, and areas of necrosis. By immunohistochemistry, all tumors demonstrated evidence of myogenic differentiation, with focal or diffuse positivity for one or more markers (desmin, h‐caldesmon, smooth muscle actin). However, none of the cases displayed diagnostic features of leiomyosarcoma or other known pathologic entities. Molecular studies revealed recurrent fusions involving *FGFR1* (*n* = 3), *FGFR2* (*n* = 1), and *FGFR4* (*n* = 2), retaining the kinase domain. Additional recurrent genomic alterations included *TP53* mutations, *CDKN2A* homozygous deletions, and *RB1* alterations. Most patients followed an aggressive clinical course, including metastases and disease‐related mortality. These findings expand the spectrum of sarcomas driven by *FGFR* gene fusions, underscoring the importance of molecular testing for accurate diagnosis and potential targeted therapy in high‐grade sarcomas with myogenic features.

## Introduction

The wide application of genomic testing in clinical practice has led to the identification of novel gene fusions as key oncogenic drivers in various subtypes of bone and soft tissue tumors. Among these, fusions involving members of the fibroblast growth factor receptor (FGFR) family – comprising *FGFR1, FGFR2*, *FGFR3*, and *FGFR4* – have emerged as recurrent molecular alterations in several rare mesenchymal neoplasms, including soft tissue chondroma [1], calcified chondroid mesenchymal neoplasm (CCMN) [2, 3, 4, 5], phosphaturic mesenchymal tumor [6], and gastrointestinal stromal tumors (GISTs) [7]. In addition, *FGFR* fusions have been recently described in tumors exhibiting an infantile fibrosarcoma (IFS)‐like morphology, suggesting that kinase fusion‐driven spindle cell neoplasms may be associated with a broader genetic spectrum [8]. Despite these advances, the incidence and clinicopathologic features of sarcomas driven by *FGFR* gene rearrangements remain poorly understood. In this study, we perform a comprehensive investigation of the presence of *FGFR* fusions among a wide spectrum of sarcomas with well‐characterized pathologic features and genomic landscape. The goals of this work were twofold: first, to better characterize if these gene rearrangements may define a potential novel sarcoma subtype, and second, to investigate the occurrence of *FGFR* fusions as passenger alterations among known sarcoma entities with well‐defined genetic drivers.

## Materials and methods

### Case selection and study cohort

After approval by the Institutional Review Board (IRB), all study subjects provided written informed consent to the use of their genomic data for research (IRB# 12‐245). Triggered by an index case of a retroperitoneal unclassified spindle cell sarcoma with myogenic differentiation harboring an *FGFR1* gene fusion, we investigated our https://www.cbioportal.org (2014–2025) for sarcoma cases associated with *FGFR1‐4* gene fusions. Previously described bone and soft tissue tumor entities associated with recurrent *FGFR* fusions were excluded [1, 2, 3, 4, 5, 6, 7, 8]. We first selected unclassified spindle cell sarcomas, not otherwise specified (NOS), that did not fit into any known pathologic category, in which the *FGFR* fusions may represent the driver alteration, thus defining a novel sarcoma entity. Clinical history, pathologic features, immunohistochemical results, and outcome data were retrieved from pathology reports and chart review. Additionally, we also sought to investigate the presence of co‐occurring *FGFR* fusions as secondary genetic alterations among well‐known sarcoma histotypes driven by other/known genomic events.

### Genomic analysis

All cases were tested using the institutional targeted sequencing platform (MSK‐IMPACT™), a comprehensive molecular assay that involves hybridization capture and deep sequencing of all exons and selected introns of 341–505 oncogenes and tumor‐suppressor genes, allowing the detection of point mutations, small and large insertions, deletions, and rearrangements. In addition, the assay captures >1,000 intergenic and intronic single‐nucleotide polymorphisms, interspersed homogenously across the genome, aiding the assessment of genome‐wide copy number. The assay uses paired tumor/normal DNA for sequencing and analysis. Details of the MSK‐IMPACT assay have been previously published [9]. The tumor mutational burden (TMB) and fraction of genome altered (FGA) were also derived from MSK‐IMPACT data. TMB was determined by calculating the ratio of nonsynonymous mutations to the total number of base pairs sequenced in each sample. The FGA was measured as the proportion of the genome exhibiting a log2 copy‐number gain greater than 0.2 or a loss less than 0.2, relative to the total size of the genome profiled for copy‐number variations. Somatic variants were called by Mutect2 and annotated by Annovar [10], and the allele specific copy number variants were called by FACETS [11]. The genomic alterations were analyzed using R package ‘ComplexHeatmap’ version 2.15.4 [12]. The segmented copy number profiles were used to generate arm level copy number changes using ASCETS package [13]. In most cases, Archer® FusionPlex® Sarcoma Panel (Archer, Boulder, CO, USA) was applied for gene fusion confirmation, which is a custom amplicon‐based next generation sequencing assay using standard protocols, as previously described [14].

## Results

### Demographics and clinical information

A total of six unclassified sarcomas (high‐grade sarcoma, NOS) harboring *FGFR* fusions were selected from the MSK‐IMPACT database. All patients except one were female, with a median age at diagnosis of 66.5 years (mean: 68.5; range: 56–90 years). The tumor sizes ranged from 7.5 to 17.5 cm (mean of 12 cm). The uterus was the most common anatomic site (*n* = 3), followed by the retroperitoneum (*n* = 2), and paraspinal muscle (*n* = 1). The clinical features are summarized in Table 1.

**Table 1:** Clinicopathological features of the *FGFR*‐fusion positive high‐grade sarcomas with myoid differentiation

Case no.	Age/Sex	Gender	Site	Size	Mitoses per 10 HPF	Necrosis	Treatment	Outcome	LR/DM	FU (months)
1	90/F	Female	Uterus	7.5	43	Present	TAHBSO	LFU	No	1
2	72/F	Female	Retroperitoneum	10.8	NA	NA	Liposomal doxorubicin*	DOD	DM (lung, pleura) at diagnosis	3.5
3	66/M	Male	Retroperitoneum	14	NA	Present	Wide excision	AWD	LR at 85 and 128 months	132
4	67/F	Female	Uterus	10.5	26	NA	TAHBSO; doxorubicin and trabectedin for metastatic disease	AWD	LR, DM (lungs) at 7 months	10
5	60/F	Female	Right paraspinal	17.5	18	Present	Wide excision	AWD	LR at 31 months	34
6	56/F	Female	Uterus	NA	14	Present	TAHBSO	DOD	LR at 4 months	14

AWD, alive with disease; DM, distant metastases; DOD, died of disease; LFU, lost follow‐up; LR, local recurrence; TAHBSO, total abdominal hysterectomy bilateral salpingo‐oophrectomy.

* Presented with metastatic disease.

### Histopathologic and immunohistochemical findings

**Figure 1:**
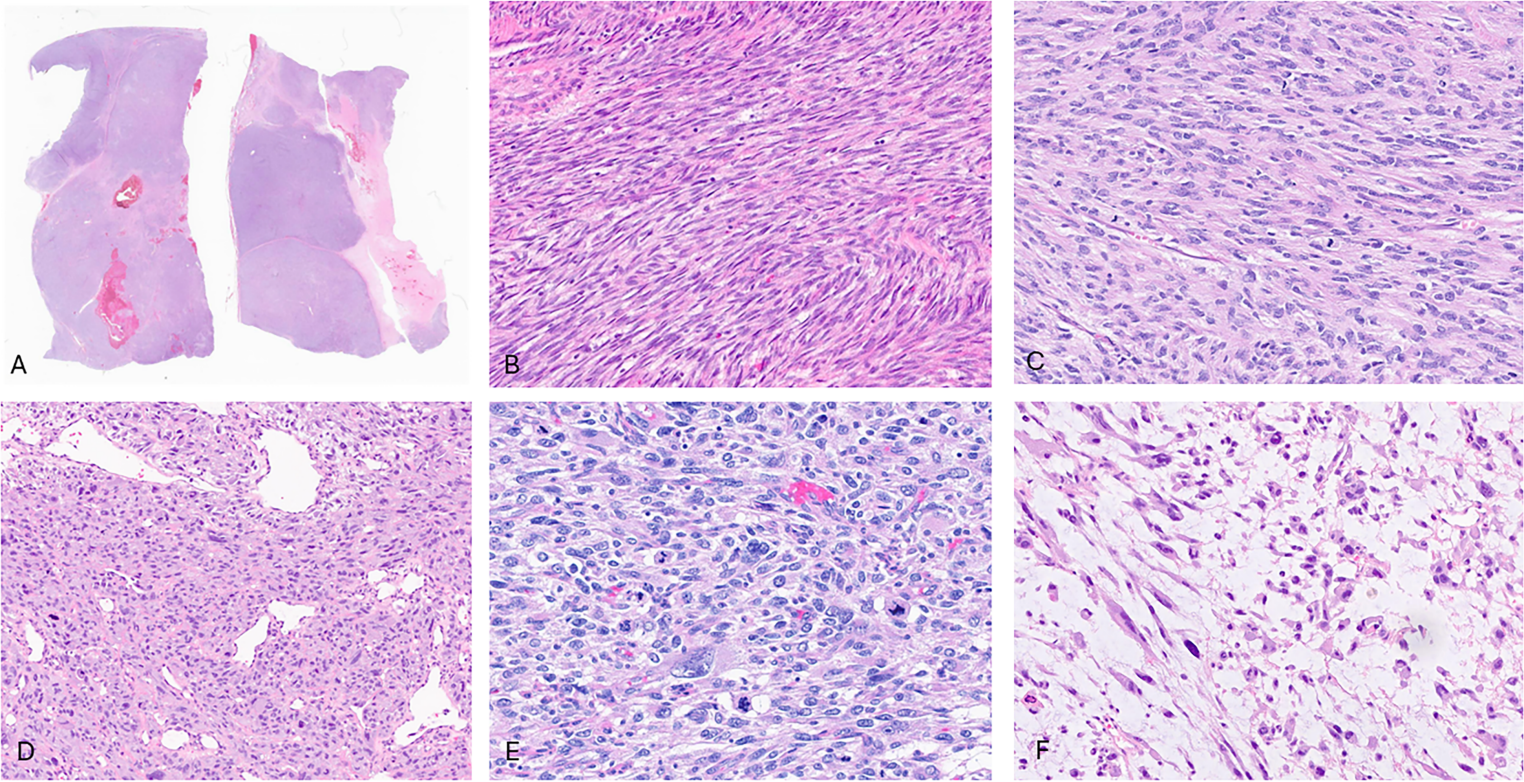
Histology of high‐grade sarcoma with myogenic differentiation and *FGFR* fusion. (A) Low power view showing well circumscribed borders and multilobular architecture with areas of geographic necrosis and hemorrhage (case 3). (B, C) The spindle cells are arranged in fascicular growth (cases 6 and 3; ×200 magnification). (D) Some tumors have overt cytologic atypia with marked nuclear pleomorphism (case 2; ×200 magnification) (E) and frequent mitoses (case 1; ×400 magnification). (F) Areas of myxoid stroma are present (case 2; ×400 magnification).

**Figure 2:**
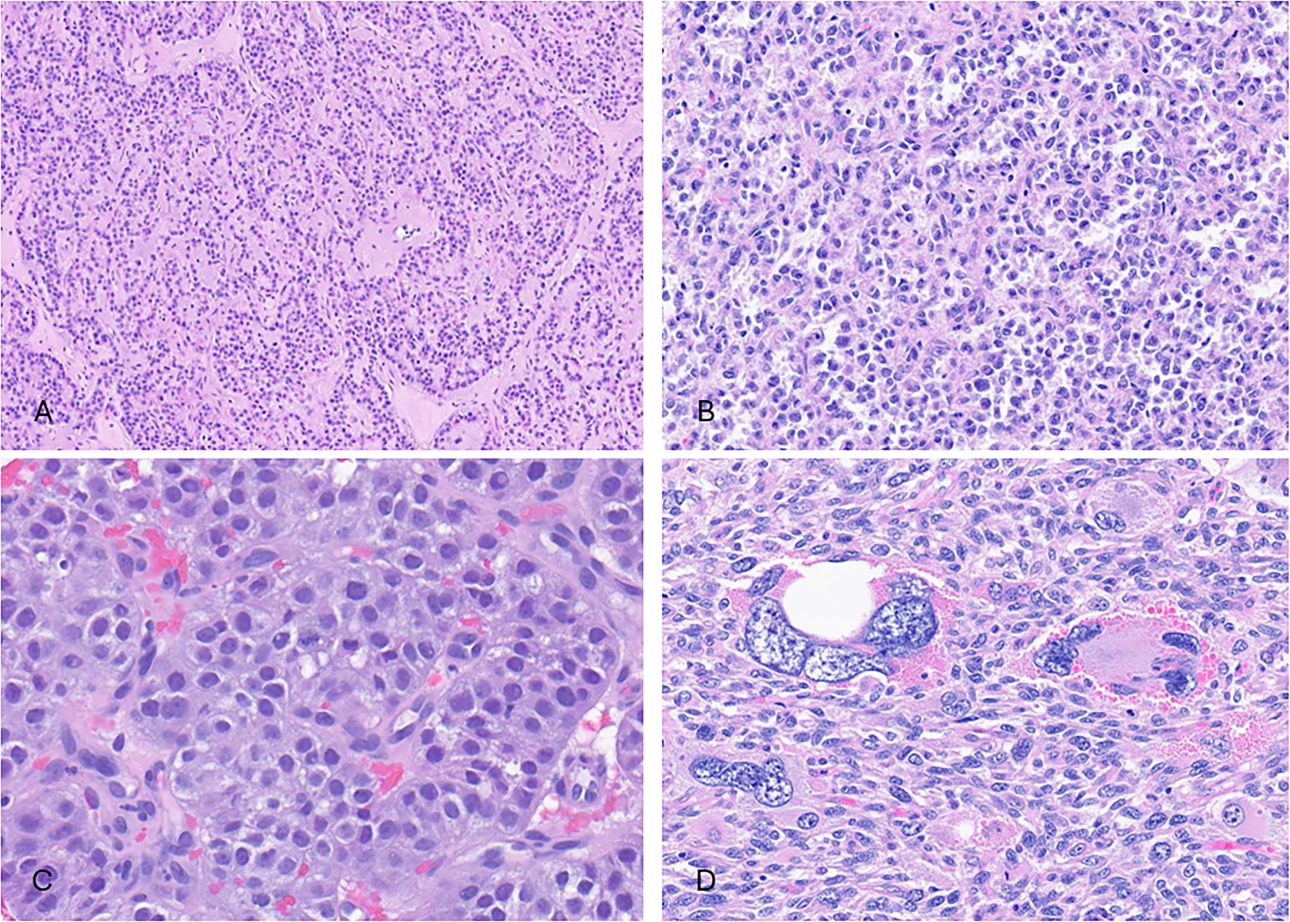
Morphologic appearance of one of the *FGFR* fusion high‐grade sarcomas with myogenic differentiation. (A–C) Epithelioid cells arranged in trabeculae, nests, cords, and alveolar‐like structures with relatively uniform cytology (case 4). (D) Multinucleated giant cells are seen in one case (case 1).

At low power, the tumors showed well‐demarcated borders, with a multinodular and solid architecture (Figure 1A). All except one case (case 4) were characterized by a spindle cell morphology arranged in intersecting fascicles (Figure 1B,C). Most tumors showed a relatively monomorphic cytology; however, a variable degree of nuclear pleomorphism was present (Figure 1D,E). The tumors showed a moderate amount of fibrillary eosinophilic to amphophilic cytoplasm. An intervening collagenous stroma was present in most cases, with one case displaying myxoid stromal changes (case 2; Figure 1F). In one case (case 4), the tumor showed a distinctive monomorphic epithelioid phenotype, arranged in nests, cords, trabeculae, and pseudo‐alveolar structures (Figure 2A–C). Scattered giant tumor cells were seen in one case (case 1; Figure 2D). Mitotic activity was high in all cases, ranging from 14 to 43 mitoses per 10 high‐power fields (HPFs) (mean, 25/10 HPFs). Atypical mitotic figures were readily seen. Necrosis was present in four cases.

**Figure 3:**
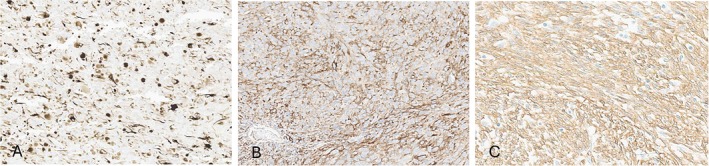
Immunohistochemical staining results of (A) desmin (case 1), (B) h‐caldesmon (case 3), and (D) smooth muscle actin (case 6).

Immunohistochemically, all cases showed positivity for at least one smooth muscle marker, in keeping with myogenic differentiation. All except one case were positive for desmin, with an additional three cases being positive for h‐caldesmon (3/3) and SMA (3/5) positivity (Figure 3). One case (case 6) was positive for all three markers. Detailed immunohistochemical results were summarized in Table 2.

**Table 2:** Key immunohistochemical and molecular findings of the study group

Case no.	SMA	Desmin	Caldesmon	Other IHC findings	Fusion	*TP53* mutation	*CDKN2A* homozygous deletion
1	+	++	NA	Positive (patchy): CD117, PRAME Positive (very focal): HMB45, panCK, EMA, claudin4 Negative: myogenin, Melan‐A, DOG1, SOX10, ER, PR	FGFR1 (ex17)::ERC1 (ex9) (Archer and IMPACT)	p.D259Y (c.775G>T)	No
2	NA	+	+++	Positive (weak, diffuse): WT1 Negative: myogenin, ER, PR, Myo‐D1, S100	FGFR1 (ex17)::HOOK3 (ex5) (Archer and IMPACT)	p.L344Q (c.1031 T>A)	No
3	−	−	+++	Positive (focal): CD34 Negative: S100, EMA, CDK4, DOG1, CD117, panCK, SOX10, S100 Others: Ki67 10–15%	FGFR1 (ex17)::HOOK3 (ex12) (Archer and IMPACT)	No	Yes
4	+++	+	NA	Positive (focal): CD10, ER, cyclin D1, TLE1 Negative: myogenin, PR, NOR1, PLAG1, inhibin, calretinin, FOXL2, CK‐pan, SOX10 Others: ARID1A, BAF47, BRG1, BRM retained	FGFR2 (ex17)::WRN (ex3) (Archer and IMPACT)	No	Yes
5	++	++	NA	Positive (patchy): CD34, EMA, MDM2 Negative: myogenin, S100, CD31, CD117, DOG1, SOX10, HMB45, CD45, ALK1, Syn, Chromogranin, STAT6, BCOR, MUC4, MNF116 Others: Ki‐67 50%. INI1, BRG‐1 intact	FGFR4 (ex17)::SH3PXD2B (ex2) (IMPACT)	Deletion	No
6	+++	+++	+++	Positive: CD10 Negative: BCOR and ALK1	FGFR4‐intragenic (breakpoint within exon 13, including kinase domain) (IMPACT)	No	Yes

+, focal; ++, patchy; +++, diffuse; NA, not available.

### Genomic findings

**Figure 4:**
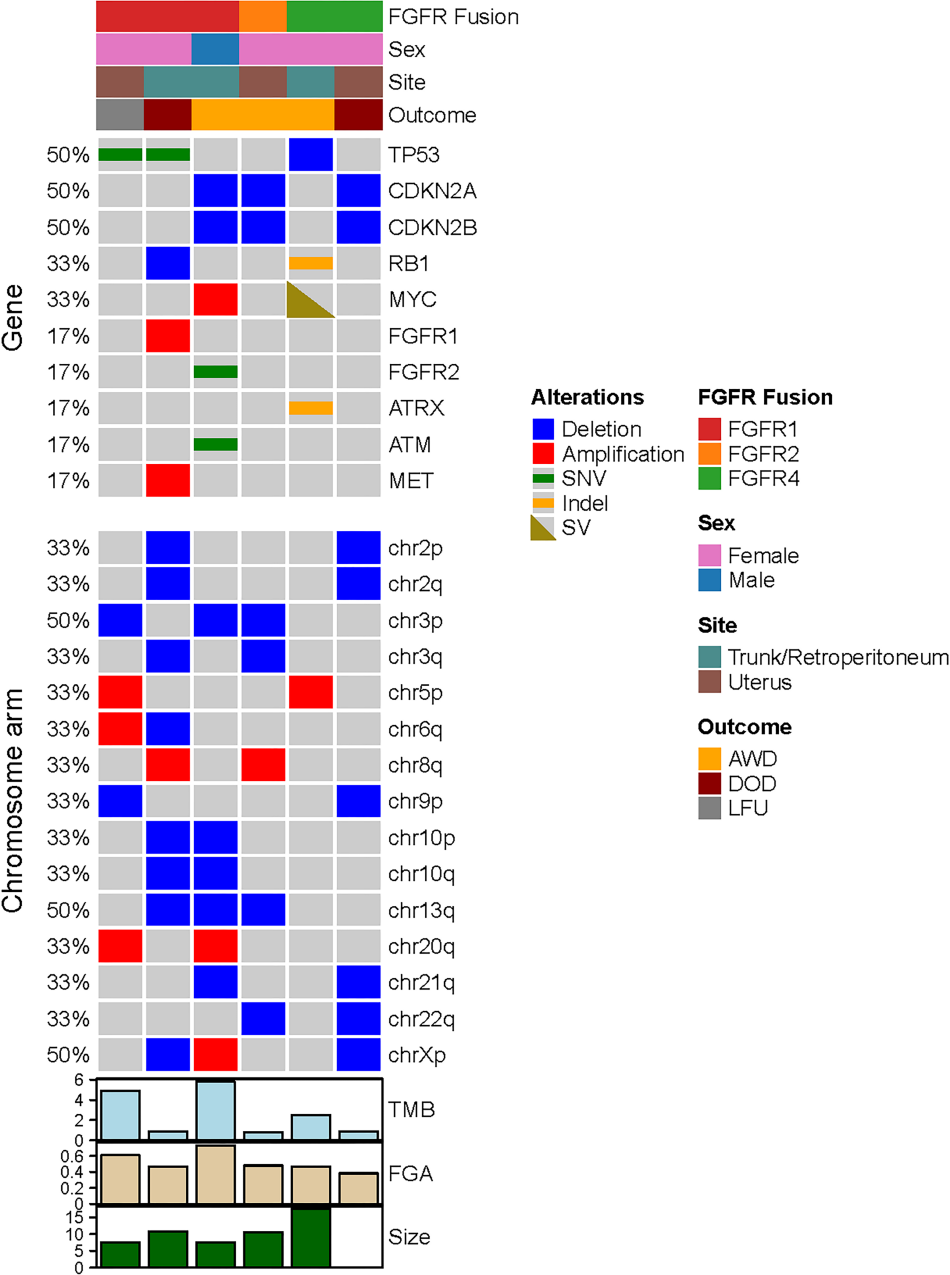
Oncoplot of molecular and clinicopathological findings in sarcoma with FGFR fusion, showing genomic alterations in selected genes and recurrent arm‐level copy number changes.

**Figure 5:**
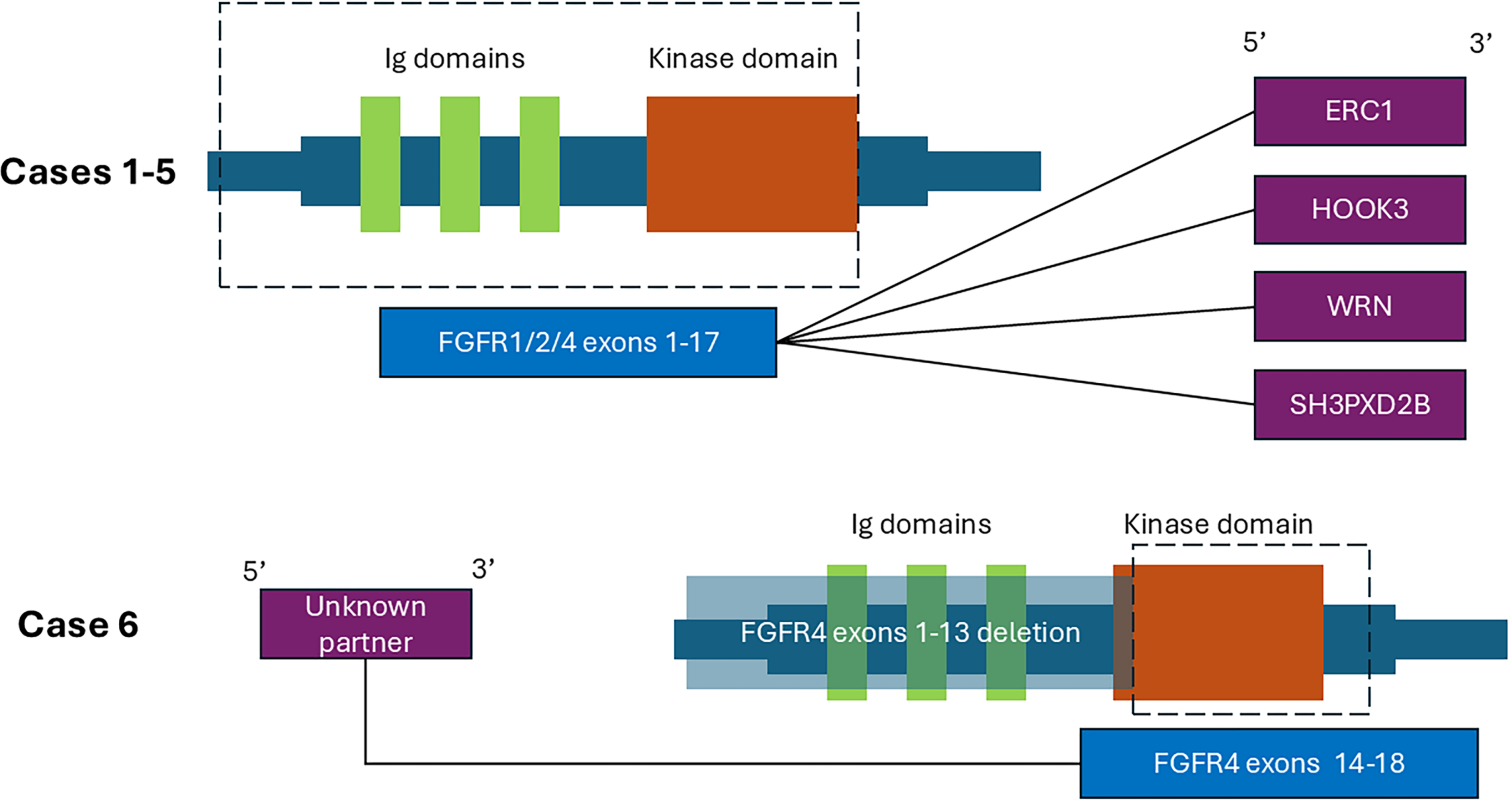
Schematic diagram of FGFR fusions in our cohort. In cases 1–5, FGFR genes were included as 5′ partner fused with different genes including *ERC1*, *HOOK3*, *WRN*, and *SH3PXD2B*. The breakpoint was consistently located after exon 17, thus retaining the kinase domain. In case 6, the *FGFR4* gene was present as 3′ partner, also retaining the kinase domain at exons 14–18.

The molecular features were summarized in Table 2 and Figure 4. Half of the cases (3/6) harbored *FGFR1* fusions, with one case showing *FGFR2* and two cases *FGFR4* fusions. All the breakpoints in *FGFR1*/*FGFR2*/*FGFR4* genes were located at exon 17 except one at exon 13, with all retaining the kinase domain in the predicted fusion oncoprotein (Figure 5). In four cases, the fusion transcript was detected by both IMPACT and Archer. In two cases, the *FGFR4* fusions were detected by IMPACT only but not by Archer. In both cases, the fusion was predicted by IMPACT to be in‐frame and included the kinase domain of FGFR4. Specifically, in case 5, the *FGFR4::SH3PXD2B* fusion resulted from an inversion, with *FGFR4* exons 1–17 being fused to *SH3PXD2B* exons 2–13. Similarly, the IMPACT analysis in case 6 detected an *FGFR4* rearrangement with breakpoint within exon 13 and including the kinase domain. In a retrospective review, the primer set for the Archer gene panel does not cover exons 17 and 13 of *FGFR4* gene, respectively, thus leading to a false‐negative result. Therefore, these two cases are expected to produce a functional fusion transcript.

By MSK‐IMPACT, additional secondary events were identified, including *TP53* alterations (*n* = 3), *CDKN2A* homozygous deletions (*n* = 3), *RB1* alterations (*n* = 2), and *MYC* alterations (*n* = 2) (Table 2, Figure 4). The presence of *TP53* alterations and *CDKN2A* deletions was mutually exclusive, while *RB1* gene alterations co‐occurred with *TP53* mutations. In addition, all cases had multiple copy number alterations, with a high FGA (median: 47.53%). However, the tumor mutation burden (TMB) was low in all cases (<10 mt/mb), with a median of 1.7 mt/mb. Recurrent arm‐level copy number changes included amplifications in 5p, 8q, and 20q, and deletions in 2p, 2q, 3p, 3q, 9p, 10p, 10q, 13q, 21q, and 22q.

### Clinical management and outcome

All except one patient presented with localized disease and underwent definitive resection with negative margins, either a wide local excision or a total hysterectomy with bilateral salpingo‐oophorectomy. The remaining patient received palliative liposomal doxorubicin chemotherapy due to surgically unresectable disease with widespread distant metastases, including lung and pleura (case 2). Follow‐up was available in five patients with a median of 10.5 months (range 7–132 months). Most patients followed an aggressive clinical course. All five patients with available follow‐up developed local recurrence. In addition to the patient presenting with metastatic disease, one additional patient developed distant metastasis to bilateral lungs, 7 months after diagnosis (case 4). This patient was currently on chemotherapy with doxorubicin and trabectedin. At last follow up, two patients succumbed of disease, and three patients were alive with disease. None of the patients were given FGFR inhibitors.

### *FGFR* fusions as secondary genetic alterations in common sarcoma types with known driver events

We identified 12 cases with *FGFR* fusions across a diverse spectrum of established sarcoma subtypes, as detailed in Table 3. These included osteosarcomas (*n* = 3), intimal sarcomas (*n* = 2), and single cases of dedifferentiated liposarcoma, myxoinflammatory fibroblastic sarcoma, spindle cell/sclerosing rhabdomyosarcoma, embryonal rhabdomyosarcoma with anaplasia, undifferentiated pleomorphic sarcoma (UPS), and synovial sarcoma. These cases were characterized by either typical morphologic features, immunoprofile and/or displayed their pathognomonic genomic alterations (i.e., *MDM2/CDK4* amplification, *SS18* fusion, etc.) for their respective diagnosis. *FGFR1* fusions were the most common (7/12), followed by *FGFR3* (*n* = 3) and *FGFR2* (*n* = 2), with all fusions predicted to be in‐frame and retaining the kinase domain, suggesting functional significance. The breakpoints were located at either exon 17 (*n* = 5) or exon 18 (*n* = 5) if the *FGFR* genes were the 5′ partner and located at exon 9 (*n* = 1) or exon 4 (*n* = 1) if they were the 3′ partner. In 10 cases, fusion transcripts were identified by Archer. In two cases, *FGFR* fusions were detected by MSK‐IMPACT with no confirmation by Archer. Specifically, one case of dedifferentiated liposarcoma harbored *FGFR1::TACC1* fusion and one case of synovial sarcoma found *FGFR2::CCDC46* fusion by MSK‐IMPACT, which were both predicted to be in‐frame with retained kinase domain. None of these patients were treated with FGFR inhibitors.

**Table 3:** FGFR fusion as likely secondary genetic alterations in specific sarcoma entities found in institutional database

Diagnosis (frequency)	FGFR fusion
Osteosarcoma (*n* = 3)	FGFR1 (Ex18)::RARB (In 4) [Archer] FGFR3 (Ex17)::TACC3 (Ex14) [Archer] FGFR3 (Ex18)::TACC3 (Ex9) [Archer]
Intimal sarcoma (*n* = 2)	FGFR1 (Ex17)::TACC1 (Ex7) [Archer] LSM1 (Ex2)::FGFR1 (Ex4) [Archer]
Dedifferentiated liposarcoma (*n* = 1)	FGFR1 (Ex18)::TACC1 (Ex7) [IMPACT]
Myxoinflammatory fibroblastic sarcoma (*n* = 1)	FGFR1 (Ex 17)::ZBTB47 (Ex 2) [Archer]
Composite hemangioendothelioma (*n* = 1)	HSPG2 (Ex74)::FGFR1 (Ex 9) [Archer]
Spindle cell/Sclerosing rhabdomyosarcoma (*n* = 1)	FGFR1 (Ex17)::ANK1 (Ex18) [Archer]
Embryonal rhabdomyosarcoma with anaplasia (*n* = 1)	FGFR2 (Ex17)::NRAP (Ex8) [Archer]
Undifferentiated pleomorphic sarcoma (*n* = 1)	FGFR3 (Ex18)::JUND (Ex1) [Archer]
Synovial sarcoma (*n* = 1)	FGFR2 (Ex18)::CCDC46 (Ex11) [IMPACT]

## Discussion

Constitutive activation of various kinases through oncogenic gene fusions plays an important role in the pathogenesis of human neoplasia, spanning many different lineages and risks of malignancies. In one large pan‐cancer study, FGFRs alterations were identified in 7.1% of tumors [15], with gene amplifications and mutations being more common than gene fusions. Most *FGFR* fusion genes have been reported in cholangiocarcinoma [16], glioblastomas [17], and urothelial carcinoma [18]. In soft tissue tumors, recurrent *FGFR* fusions often involving the *FN1* gene partner were first described in phosphaturic mesenchymal tumors [6], soft tissue chondroma [1], and calcified CCMN [1, 2, 3, 4, 5]. More recently, *FGFR* fusions have also been described in three cases of pediatric mesenchymal tumors sharing histologic overlap with IFS and ‘*NTRK*‐rearranged’ spindle cell neoplasms [8]. Additionally, *FGFR* fusions have been reported in quadruple wild‐type GIST [7], uterine sarcomas [19, 20], rhabdomyosarcoma [21], and a high‐grade undifferentiated spindle cell sarcoma [22]. Mesenchymal tumors featuring *FGFR* fusions are summarized in Table 4.

**Table 4:** Mesenchymal neoplasms featuring FGFR fusion

Neoplasm	Molecular findings	Key clinicopathological features	References
Soft tissue chondroma	*FN1::FGFR1* or *FN1::FGFR2* (~20–30%)	Benign cartilaginous tumor; adults (30–60 years); acral sites (hands/feet); well‐circumscribed, lobulated hyaline cartilage with/without calcifications; rare recurrence if incomplete excision	[1]
Calcified chondroid mesenchymal neoplasm (CCMN)	*FN1::FGFR2* (~50%); *FN1::FGFR1* (~10%); other FN1::RTK fusions (e.g., *FN1::NTRK1*, *FN1::MERTK*, *FN1::TEK*; ~40%); *FGFR1::PLAG1* (rare)	Emerging entity; young adults (20–50 years); distal extremities or TMJ; small nodules with chondroid matrix, calcifications (grungy/lacy), and bland chondrocyte‐like cells in cords/lobules; low‐grade, nonaggressive (recurrence <10%); overlaps with soft tissue chondroma; no TIO association.	[2, 3, 4, 5]
Phosphaturic mesenchymal tumor (PMT)	*FN1::FGFR1* fusion (~50–60% of cases); *FN1::FGF1* fusion (~6%); FGF23 overexpression (nearly all cases)	Adults (mean age ~40–50 years); head/neck/extremities common sites; often small (<3 cm), bland spindle/ovoid cells in myxoid/hemangiopericytoma‐like stroma with grungy calcifications; causes tumor‐induced osteomalacia (TIO) in ~80–90; benign behavior but locally recurrent if incompletely excised.	[6]
Pediatric mesenchymal tumors	*FGFR1::EBF2* *FGFR1::PARD6B*	Infants (3–9 months); deep soft tissue (gluteal/pelvic/perirectal); cellular spindle/stellate cells with myxoid stroma, focal epithelioid features; low mitotic rate, no necrosis; focal CD34+; locally aggressive (recurrence possible); overlaps morphologically with infantile fibrosarcoma/NTRK tumors.	[8]
Uterine sarcoma	*FGFR1::TACC1*	Premenopausal women; uniform spindle cells in fascicles with herringbone pattern, myxoid/collagenous stroma; infiltrative, high‐grade; CD34/S100 co‐expression; aggressive (metastases/recurrence ~50%)	[19, 20]
Gastrointestinal stromal tumors (GIST)	*FGFR1::HOOK3* *FGFR1::TACC1* *FGFR2::TACC2*	Wild‐type GIST; Occur in younger patients, frequent metastasis; May act as secondary genomic alteration that contribute to multidrug‐resistance	[7]
Rhabdomyosarcoma (RMS)	*FGFR1::ANK1* *FGFR1::TACC1* FGFR1 overexpression	Young age (7–24 years); Unclassified RMS, embryonal RMS with or without anaplasia	[21]
Undifferentiated spindle cell sarcoma	*FGFR1::TACC1*	83‐year‐old man; monomorphic spindle cells arranged in intersecting fascicles; focal rare S100+; rapid recurrence with distant metastasis	[22]
High‐grade sarcomas with myoid differentiation	*FGFR1/2/4*::Various partners	High‐grade sarcomas with myoid differentiation; aggressive clinical behavior	Current cohort

**Figure 6:**
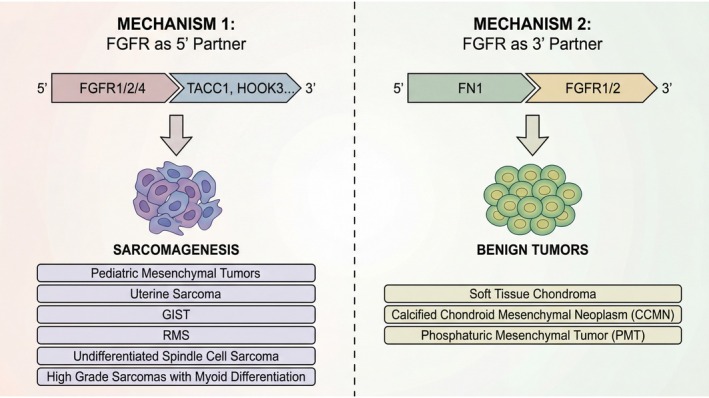
Clinical dichotomy of *FGFR* fusions based on gene topology. The position of the *FGFR* gene within the fusion transcript correlates with biologic potential. Fusions featuring *FGFR* as the 5′ partner drive malignant sarcomagenesis, whereas fusions with *FGFR* as the 3′ partner define a spectrum of benign mesenchymal neoplasms.

The FGFR family consists of four highly conserved single transmembrane tyrosine kinase receptors (FGFR1‐4) that are essential to physiologic processes such as cell proliferation, survival, and differentiation [23]. Similar to most other receptor tyrosine kinases (RTKs), FGFR comprises an extracellular ligand‐binding domain and a cytoplasmic conserved tyrosine kinase domain. However, in contrast to most other RTKs, there are two mechanisms generating oncogenic FGFR fusions, both resulting in ligand‐independent dimerization and constitutive activation of the FGFR kinase signaling pathway (Figure 6). The first includes the FGFR as a 5′ partner, in which the N‐terminal domain, including the kinase domain of FGFR1‐4 (exons 11–18), is fused to a 3′ partner containing a constitutive dimerization/oligomerization motif [24, 25]. This fusion transcript is encountered in pediatric kinase fusion‐positive mesenchymal tumors [8], uterine sarcoma [20], GIST [7], and rhabdomyosarcoma [21]. This fusion structure was also found in all cases of the current cohort of high‐grade sarcomas with myogenic differentiation, including *FGFR1::ERC1*, *FGFR1::HOOK3*, *FGFR2::WRN*, and *FGFR4::SH3PXD2B*. Among these fusion partners, the coiled‐coil domains of HOOK3 and the oligomerization motifs of SH3PXD2B may facilitate spontaneous dimerization or clustering of the fusion proteins, promoting autophosphorylation and sustained kinase activity. This ligand‐independent activation of FGFR kinases results in the persistent activation of downstream signaling pathways, such as MAPK/ERK and PI3K/AKT, which are critical regulators of cell proliferation, survival, and migration. For the second mechanism, the C‐terminal of the FGFR protein is retained in the fusion protein, which is fused to the N‐terminal of FN1. The FN1 partner functions as a strong promoter, with the resulting fusion protein facilitating secretion and multimerization, and constitutive activation of the FGFR kinase signaling pathway. This alternative mechanism of fusion is detected within the spectrum of benign mesenchymal neoplasms, such as soft tissue chondroma, CCMN, and PMT [6]. In these cases, the 3′ end of the FGFR gene (breakpoint typically occurs at exons 5–9) includes the transmembrane domain (exon 10) and the entire tyrosine kinase domain (exons 11–18) [2].

Strikingly, the topologic orientation of the *FGFR* gene within the fusion construct strongly correlates with the histotype and clinical behavior, as 5′‐*FGFR* fusions predominantly drive sarcomas, whereas 3′‐*FGFR* fusions characterize benign neoplasms. This profound clinical divergence likely stems from differences in the spatial distribution and amplitude of their kinase signaling. When *FGFR* acts as the 5′ partner, fusion to 3′ coiled‐coil domains (e.g., *HOOK3*, *TACC1*) drives robust, ligand‐independent oligomerization. This intense constitutive activation – potentially exacerbated by the fusion protein evading normal lysosomal degradation via cytoplasmic mislocalization – overwhelms intrinsic feedback loops and drives relentless downstream signaling, culminating in cellular dedifferentiation and sarcomagenesis [26]. Conversely, in 5′‐*FN1* fusions, fibronectin likely tethers the kinase to the secretory pathway or the extracellular matrix [27]. We postulate that this spatial confinement restricts the kinase from accessing critical intracellular substrates, resulting in a lower‐amplitude signal. Furthermore, while native *FGFR* promoters (active in 5′ fusions) may link to cellular plasticity, the strong *FN1* promoter primarily drives localized matrix deposition and paracrine/endocrine signaling (e.g., FGF23). Thus, rather than cell‐autonomous malignant transformation, FN1‐driven fusions create a localized, hyperactive matrix‐modulating loop, leading to histologically benign neoplasms. Ultimately, fusion topology does not merely act as an ‘on‐switch’ for *FGFR*, but fundamentally dictates the spatial context and oncogenic trajectory of the signal [28].

Kinase fusion‐positive spindle cell neoplasms represent an emerging, molecularly defined subset, sharing a spectrum of morphologies and immunoprofiles [29]. Recent studies have implicated a wide range of kinases, including *NTRK1*, *NTRK2*, *NTRK3* [30, 31, 32, 33], *BRAF* [34, 35, 36], *RAF1* [37, 38], *RET* [39], and *LTK* [40]. The phenotypic diversity includes mostly three architectural patterns, such as lipofibromatosis‐like neural tumors, IFS‐like and MPNST‐like [41], with common, but inconsistent, co‐expression of CD34 and S100 markers. In a recent study, *FGFR1* fusions have been found in three infants with IFS‐like tumors, arising in the gluteal and pelvic deep soft tissues [8]. Additionally, two cases of uterine sarcomas with *FGFR1* fusion were reported sharing a FS‐like phenotype with moderate nuclear atypia and co‐expression of CD34 and S100, likely fitting among the spectrum of kinase fusion sarcomas with high‐grade morphology [19, 20]. In contrast, the three uterine tumors in the current series, harbored *FGFR1, FGFR2*, and *FGFR4* fusions, diverse histologic features and expression of smooth muscle markers, suggesting distinct pathogenesis. Another case report of an *FGFR1*‐rearranged high‐grade sarcoma occurring in the thigh of an 83‐year‐old man showed an FS‐like morphology and expression of S100, while CD34 and all three muscle markers were negative [22].

The histologic features seen in the current series did not fit with any well‐defined pathologic entities, including the morphologic spectrum of kinase fusion positive spindle cell neoplasms. Instead, tumors showed a high‐grade mostly spindle cell morphology with variable nuclear pleomorphism, increased mitotic activity and necrosis. Notably, all tumors showed expression of smooth muscle markers, including desmin in all except one case, in keeping with a high‐grade sarcoma with myogenic differentiation. Of note, none of the cases fulfilled the diagnostic criteria for leiomyosarcoma, lacking both the distinctive architectural pattern of perpendicularly intersecting long fascicles, as well as the typical cytologic features, such as fibrillary eosinophilic cytoplasm and elongated, cigar‐shaped nuclei. We have considered the possibility of a dedifferentiated leiomyosarcoma; however, no well‐differentiated component was identified in any of the extensively sampled cases. Moreover, no skeletal muscle markers were expressed to suggest a rhabdomyosarcoma diagnosis. Especially for the uterine location, another consideration was that of a malignant PEComa, but no expression of melanocytic markers, such as HMB45 or Melan‐A, was detected. In the retroperitoneum, another major differential diagnosis was dedifferentiated liposarcoma; however, there was no well‐differentiated liposarcoma component nor *MDM2* and *CDK4* gene amplifications. The possibility of a high‐grade UPS was also considered, but the relatively monomorphic phenotype and consistent smooth muscle expression argued against.

Our findings suggest that *FGFR* fusions may represent the driver alteration in a subset of high‐grade sarcomas with myogenic differentiation, adding this phenotype to the growing list of tumor types led by this oncogenic fusion. *FGFR1* fusions were the most common variant, but regardless of the gene involved, the kinase domain retained in the fusion was provided by the N‐terminal of the FGFR protein. The same mechanism is shared by all the other soft tissue sarcomas and kinase fusion spindle cell neoplasms harboring this fusion [8], in contrast to the opposite orientation of *FN1::FGFR1* fusions found in the benign mesenchymal neoplasms [2]. In addition, our study sheds light on the incidence and sarcoma histotypes in which *FGFR* fusions were detected likely as passengers or secondary events in otherwise well‐characterized sarcoma entities. The morphologic spectrum of this latter group was quite broad, but appeared enriched in genomically complex entities, such as osteosarcoma, UPS, etc. The most common fusion involved *FGFR1*, as the 5′ partner. Notably, the *FGFR3::TACC3* fusion identified in two cases of osteosarcoma aligns with recent bioinformatics and functional studies confirming their role in enhancing invasiveness and metastasis [42, 43]. The functional significance was further validated by an *in vivo* study that demonstrated the success of induced pluripotent stem cell‐generated cytotoxic T lymphocytes targeting *FGFR3::TACC3* to inhibit growth and metastasis in a nude mouse osteosarcoma orthotopic transplantation model [44].

Finally, the identification of *FGFR* fusions does not only impact the diagnosis and classification of these tumors, but may also represent an important druggable target. FGFR selective inhibitors targeting FGFR fusions, such as erdafitinib, pemigatinib, and futibatinib [45], have been approved by the U.S. Food and Drug Administration (FDA) to treat patients with urothelial cancer and cholangiocarcinoma. Our findings underscore the importance of integrating molecular testing into the diagnostic workup of unclassified high‐grade sarcomas and provide new insights into the biology of this emerging sarcoma subset.

In summary, we describe the clinical, histopathologic, and molecular features of six cases of high‐grade sarcoma with myogenic differentiation harboring *FGFR* fusions. Patients followed an aggressive clinical course, with local recurrences and distant metastases. As these tumors were initially deemed unclassified, not fitting any known pathologic categories, it is likely that the *FGFR* fusions may define a novel subset of high‐grade sarcomas with myogenic lineage. In addition, the presence of *FGFR* fusions highlights their potential as therapeutic targets, in using FGFR inhibitors as a promising strategy for these patients. Future larger studies with integrated genomic alterations may further support the accurate classification of these lesions. Additional functional studies are also warranted to elucidate the precise mechanisms by which these fusions contribute to sarcomagenesis and to explore their therapeutic implications.

## Author contributions statement

MCFY performed data retrieval and genomic analysis, reviewed histology and wrote the manuscript. CS reviewed the genomic data. CRA conceived and supervised the study, reviewed the histology and wrote the manuscript. All authors reviewed and approved the manuscript.

## Data Availability

The data supporting the findings of this study are available within the article, or from the corresponding author upon reasonable request.
